# *Escherichia coli* outer membrane protein F (OmpF): an immunogenic protein induces cross-reactive antibodies against *Escherichia coli* and *Shigella*

**DOI:** 10.1186/s13568-017-0452-8

**Published:** 2017-07-19

**Authors:** Xiao Wang, Da Teng, Qingfeng Guan, Ruoyu Mao, Ya Hao, Xiumin Wang, Junhu Yao, Jianhua Wang

**Affiliations:** 1 Key Laboratory of Feed Biotechnology, Ministry of Agriculture, Beijing, 100081 People’s Republic of China; 2 College of Animal Science and Technology, Northwest A&F University, Yangling, Shaanxi 712100 People’s Republic of China; 3 Gene Engineering Laboratory, Feed Research Institute, Chinese Academy of Agricultural Sciences, Beijing, 100081 People’s Republic of China

**Keywords:** *Escherichia coli*, Recombinant OmpF, Immune protection, Vaccine, Mice

## Abstract

Diarrhea caused by pathogenic *Escherichia coli* (*E. coli*) is one of the most serious infectious diseases in humans and animals. Due to antibiotics resistance and the lack of efficient vaccine, more attention should be paid to find potential versatile vaccine candidates to prevent diseases. In this study, the sequence homology analysis indicated that OmpF from *E. coli* CVCC 1515 shares a high identity (90−100%) with about half of the *E. coli* (46.7%) and *Shigella* (52.8%) strains. Then the recombinant OmpF was supposed to be developed as a versatile vaccine to prevent *E. coli* infection. OmpF was expressed in *E. coli* BL21 (DE3) using the auto-induction method. The recombinant OmpF (rOmpF) protein had an average molecular weight of 40 kDa with the purity of 90%. Immunological analysis indicated that the titers of anti-rOmpF sera against rOmpF and whole cells were 1:240,000 and 1:27,000, respectively. The opsonophagocytosis result showed that 72.21 ± 11.39 and 11.04 ± 3.90% of bacteria were killed in the rOmpF immunization and control groups, respectively. The survival ratio of mice immunized with rOmpF ranged between 40 and 60% as observed within 36 h after challenge, indicating mice were partially protected from *E. coli* CVCC 1515 infection. The expressed rOmpF protein induced an effective immune response, but only provide a weak protection against pathogenic *E. coli* CVCC 1515 and a small reduction in *E. coli* CICC 21530 (O157:H7) excretion in a mouse infection model. Native forms of the OmpF antigen may be studied for immunogenicity and potential protective efficacy.

## Introduction

Bacterial diarrhea caused by enterotoxigenic *Escherichia coli* is the main infectious disease in humans and animals worldwide (Johnson et al. 2010). Enterotoxigenic *E. coli* is transmitted by food or water contaminated with animal or human feces. The *E. coli* CVCC 1515 (O149:K91 and K88ac) strain, a predominant serotype, occurred more frequently in neonatal and postweaning pigs (Noamani et al. 2003; Maynard et al. 2003). Urease-positive *E. coli* CVCC 1515 was responsible for over 90% of cases of post-weaning diarrhea in recent outbreaks in Canada, which leading to substantial economic losses (Noamani et al. 2003). None of the attempted solutions to the problem of post-weaning diarrhea due to enterotoxigenic *E. coli* in pigs has been consistently effective. Another enteric pathogen *E. coli* CICC 21530 (O157:H7) strain is major cause of food-borne diarrheal disease, and can produce large quantities of one or more related potent toxins that cause severe damage to the lining of the intestine. Close to 75,000 cases of *E. coli* O157:H7 infection with 2–10% deaths are now estimated to occur annually in the United States (Perna et al. 2001; Vali et al. 2004). Although antibiotics and vaccines are currently available to prevent *E. coli*-induced diarrheas, antibiotics residues may pose severe health hazards in human and there are few available vaccines against homologous *E. coli* challenge. Therefore, more attention should be paid to find potential versatile vaccine candidates to prevent diseases induced by *E. coli*.

Outer membrane proteins (OMPs), exposed on the surface of gram-negative bacteria, are quickly recognized as extracellular foreign particles by the host immune system, thereby generating an immune response against bacterial pathogens (Osman and Marouf 2014). Highly immunogenic OMPs can thus be exploited as vaccine candidates against several bacterial species such as *Chlamydia trachomatis*, *Neisseria meningitides*, *Aeromonas hydrophila* and *Edwardsiella tarda* (Pal et al. 1997; Wright et al. 2002; Khushiramani et al. 2012; Yadav et al. 2014; Okamura et al. 2012). Among OMPs, the outer membrane protein F (OmpF) and OmpC are the two most common porins that make 2% of the total cellular protein, and OmpF is the best-characterized porin protein in terms of structural and functional characteristics (Williams et al. 2000). OmpF consists of 16 antiparallel β-strands forming a barrel embedded in the membrane and displays eight domains of the surface antigen at the N-terminal extracellular domain (http://www.uniprot.org/) (Williams et al. 2000). Several attempts have been made to evaluate the OmpF immunogenicity of gram-negative bacteria. Secundino et al. showed that OmpF of *Salmonella typhi* could induce a sustained, lifelong and specific bactericidal antibody response (Secundino et al. 2006). Synthetic peptides representing certain epitopes of the OmpF of *Pseudomonas aeruginosa* have been reported to confer protection against *P. aeruginosa* infections in a mouse model (Hughes and Gilleland 1995). Liu et al. demonstrated that the recombinant OmpC and OmpF proteins from *E. coli* stimulated strong immunoglobulin G (IgG) antibody responses, and provided 62.5 and 87.5% protection against *E. coli* PCN033, respectively (Liu et al. 2012). Immunization with OmpF of *Yersinia pseudotuberculosis* not only resulted in production of high-avidity antibodies, but also stimulated bactericidal activity of peritoneal macrophages (Sidorova et al. 2014). Sharma et al. suggested that the OmpF epitope (66–80) in fusion with a carrier protein is a promising vaccine candidate against *A. hydrophila* (Sharma and Dixit 2015).

In the present study, the OmpF protein clusters in *E. coli*, *Shigella* and *Salmonella* were obtained from UniProtKB database and the homology was analyzed. As a conservative protein, the *ompF* gene was cloned from the genomic DNA of *E. coli* CVCC 1515 and expressed in *E. coli* BL21 (DE3) by the auto-induction method. After purification by Ni^2+^-NTA affinity chromatography, the recombinant OmpF (rOmpF) was used as an antigen to immunize mice. The protection efficiency of rOmpF vaccine was evaluated against the pathogenic *E. coli* CVCC 1515 and CICC 21530 (O157:H7) strains in vitro and in vivo.

## Materials and methods

### Bacterial strains and plasmids

Strains of *E. coli* CVCC 1515, *Salmonella enteritidis* CVCC 3377 and *Salmonella pullorum* CVCC 503 were purchased from the China Veterinary Culture Collection Center (CVCC) (Beijing, China). *Shigella dysenteriae* CMCC 51252 and *Shigella flexneri* CMCC 51571 were purchased from the National Center for Medical Culture Collection (CMCC) (Beijing, China). *E. coli* CICC 21530 (O157:H7), *Pseudomonas aeruginosa* CICC 10419 and CICC 21630 strains were purchased from the China Center of Industrial Culture Collection (CICC) (Beijing, China). *E. coli* DH5α and BL21 (DE3) strains were purchased form TransGen Biotech Co., Ltd. (Beijing, China). The pMD™ 19-T Simple and pET-28a(+) vectors with His tags were obtained from TaKaRa Biotechology Co., Ltd. (Dalian China) and Novagen (America), respectively.

### Cloning of the *ompF* gene

The primer pairs of ompF-fw-*Bam*HI: 5′-GGATCCGCAGAAATATATAACAAAGATGGC-3′ and ompF-rev-*Xho*I: 5′-CTCGAGTTAGAACTGATAAACGATACCCACA-3′ were designed according to the sequence of the *ompF* gene in *E. coli* UMNK88 (GenBank Accession No. CP002729.1) using Primer Premier 5.0. Genomic DNA was extracted from *E. coli* CVCC 1515 using a TIANamp Bacteria DNA Kit (Tiangen Biotech, Beijing, China) following the manufacturer’s instructions and used as a PCR template. The *ompF* gene was amplified and cloned into the pMD19-T Simple vector. The resultant positive pMDompF plasmid was isolated using the TIANprep Mini Plasmid Kit (Tiangen Biotech, Beijing, China), and digested with *Bam*HI and *Xho*I (NEB, Beijing, China). The digested fragment was inserted into the pET-28a(+) vector digested with same enzymes, and transformed into *E. coli* BL21 (DE3). The positive transformants were confirmed by colony PCR and DNA sequencing, respectively.

### Homological analysis of OmpF

After obtaining the *ompF* gene and amino acid sequence of *E. coli* CVCC 1515, the Basic Local Alignment Search Tool (BLAST) was used to find the local similarity between sequences of OmpF in typical *E. coli* strains (CICC 21530 (O157:H7), K12 and BL21). Then the similar protein clusters of *E. coli*, *Shigella* and *Salmonella* in the data base of uniprot uniref100 (http://www.uniprot.org/blast/) were also obtained for homology searches. MEGA5.1 software was used for the construction of a phylogenetic tree and the clusters whose size were less than 4 (*E. coli*) or 3 (*Shigella* and *Salmonella*) were omitted.

### Expression and purification of the rOmpF protein

The positive transformants were cultured for 24 h at 30 °C in ZYM-5052 auto-inducing media (300 mL in the 1 L shaking flask, 100 μg/mL kanamycin) (Studier 2005; Guan et al. 2015). The positive transformant was cultured in LB medium on a platform shaker (37 °C, 250 rpm) to an optical density at 600 nm (OD_600 nm_) of 0.4–0.6. Cells were inoculated to auto-induction media (1% inoculum density) and cultured for 24 h on a platform shaker (37 °C, 300 rpm). 1 mL of cultured cells were collected at 4, 6, 8, 10, 12, 14, and 24 h respectively by centrifugation (8000×*g*, 2 min), and analyzed by 12% sodium dodecyl sulfate polyacrylamide gel electrophoresis (SDS-PAGE). After 24 h of auto-induction, the cultured cells were harvested by centrifugation (5000×*g*, 30 min), resuspended in 50 mM Tris–HCl buffer (pH7.9, containing 5 mg of lysozyme and 5 μL of DNaseI type IV stock/g cell paste), and sonicated for 5−6 min with an Ultrasonic Crasher Noise Isolating Chamber (SCIENTZ, Ningbo Science Biotechnol Co., Ltd., China) on ice. The insoluble fractions of the cells were collected by centrifugation (14,000×*g*, 20 min), washed twice in 50 mM Tris–HCl buffer [pH 7.9, containing 1.5% (v/v) lauryldimethylamine oxide (LDAO)], and suspended in 10 mM Tris–HCl buffer [pH 7.5, containing 1 mM ethylenediamine tetraacetic acid (EDTA) and 8 M urea]. After centrifugation (14,000×*g*, 20 min), the supernatant was added into 20 mM Tris–HCl buffer [pH 7.9, containing 1 M NaCl and 5% (v/v) LDAO]. The solution was dialyzed in 20 mM Tris–HCl buffer [pH 7.9, containing 0.5 M NaCl and 0.1% (v/v) LDAO].

The rOmpF protein was purified by Ni^2+^-NTA affinity chromatography and refolded according to the previous methods (Guan et al. 2015; Saleem et al. 2012). Briefly, the cell lysis solution was loaded onto a Ni^2+^-nitriloacetate (NTA) resin column (QIAGEN, Germany), which was pre-equilibrated with 20 mM Tris–HCl buffer [pH 7.9, containing 0.5 M NaCl, 0.1% (v/v) LDAO and 40 mM imidazole]. The column was washed with 20 mM Tris–HCl buffer [pH 7.4, containing 0.5 M NaCl, 0.1% (v/v) LDAO and 500 mM imidazole]. rOmpF was then desalted with 20 mM Tris–HCl buffer [pH 7.4, containing 150 mM NaCl and 0.1% (v/v) LDAO] using a HiPrep 26/10 desalting column. All protein elutions were analyzed by 12% SDS-PAGE. The purity and yield of rOmpF protein was calculated by the Gel-Pro Analyzer™ version 6.3 (Media Cybernetics). The purified rOmpF protein was lyophilized in a freeze dryer (ALPHA 1-2 LD plus, Christ, Germany).

### Mouse immunization and challenge

Forty female SPF BALB/c mice, 6–8 weeks old, were purchased from Vital River, Beijing, China. The mice were immunized with rOmpF (20 mice) or PBS (control, 20 mice) according to the previous method reported by Reddy et al. (2010). The first injection solution consisted of 25 μg of rOmpF in sterile PBS (75 μL) and complete Freund’s adjuvant (25 μL) (Sigma-Aldrich, Inc.). Mice were hypodermically injected with antigen mixture (100 μL/mouse). The mice in the control group were immunized with PBS instead of rOmpF. Subsequent two injections containing 25 μg of rOmpF in sterile PBS (75 μL) and incomplete Freund’s adjuvant (25 μL) (Sigma-Aldrich, Inc.) were given every 2 weeks. Five days after each immunization, 10 mice were bled from the tail vein, and the serum was isolated and stored at −20 °C until use.

Two weeks after the second immunization, all the mice (40) were randomly divided into four groups as follows: (i) 10 rOmpF-immunized mice (group 1) and 10 PBS-immunized mice (group 2, control) were challenged with 10^9^ colony forming unit (CFU) *E. coli* CVCC 1515 (1 mL) by intraperitoneal injection; (ii) 10 rOmpF-immunized mice (group 3) and 10 PBS-immunized mice (group 4, control) were challenged with 10^10^ CFU *E. coli* CICC 21530 (O157:H7) (0.2 mL) by gastric tube. The mortality of mice and *E. coli* in fecal shedding of control and rOmpF immunized mice was recorded daily for 7 days.

The animal protocol for the present study was approved by the Animal Care and Use Committee of the Feed Research Institute, Chinese Academy of Agricultural Sciences (Beijing, China), and all mice involved were cared for in accordance with the institutional guidelines from the above Committee.

### Western blotting analysis of rOmpF

The SDS-PAGE was performed by loading purified rOmpF protein (about 0.1 μg) in gel for 120 min at 80 V. Subsequently, the protein was transferred to the PVDF membrane. Followed by blocking overnight with 5% BSA in TBST (25 mM Tris, 150 mM NaCl, and 0.05% (v/v) Tween-20, pH 7.4) at 4 °C, the PVDF membrane was washed three times with TBST and then incubated with the rOmpF sera (1:5000) for 2 h at room temperature. After another washing step, the membrane was incubated with secondary antibodies (Beijing CWBIO Co., Ltd.) at a dilution of 1:5000 for 2 h at room temperature. Finally, the bands were stained using BCIP/NBT solution (Beijing CWBIO Co., Ltd.) as substrate.

### Detection of specific antibodies by the indirect enzyme-linked immunosorbent assay (iELISA)

The titers or capacities of antisera against rOmpF and bacteria (*E. coli* CVCC 1515, *E. coli* CICC 21530, *S. dysenteriae* CMCC 51252, *S. flexneri* CMCC 51571, *S. enteritidis* CVCC 3377, *S. pullorum* CVCC 503, *P. aeruginosa* CICC 21630, and *P. aeruginosa* CICC 10419) were measured by iELISA (Guan et al. 2015; Hu et al. 2010). rOmpF was dissolved in coating buffer (pH 9.6, 0.015 M sodium carbonate, 0.035 M sodium bicarbonate). The 96-well plates were coated with 2 μg/mL of the rOmpF solution or 10^6^ CFU/mL bacteria solution (each well 100 μL), incubated overnight at 4 °C, and washed four times with 0.01 M PBS (containing 0.05% Tween 20). The plates were blocked for 2 h at 37 °C by adding 0.01 M PBS (containing 5% BSA), washed three times, and then incubated with serial dilutions of mice serum at 37 °C for 1.5 h. After washing as above, 100 μL of horseradish peroxidase (HRP) conjugated goat anti-mouse IgG (1:5000) was added into each well and incubated for 30 min at 37 °C. The plates were washed three times again. 100 μL of 3, 3′, 5, 5′-tetramethylbenzidine (TMB) was added to each well and incubated for 20 min in the dark at room temperature. Finally, the color reaction was stopped by adding 2 M H_2_SO_4_ (50 μL/well). The absorbance of each well at 450 nm was determined by an automatic ELISA plate reader (Perlong Medical, Beijing). The result was considered as positive when the ratio of the test group and negative control group was greater than 2.1 (Lunin et al. 2009; Xu et al. 2011).

### Opsonophagocytosis assay

Murine peritoneal macrophages cells were isolated as previously described and adjusted to 4 × 10^6^ CFU/mL (Guan et al. 2015; Zhang et al. 2008). Briefly, after incubation with 100 μL of anti-rOmpF sera or anti-PBS sera at 37 °C for 30 min, 400 μL of *E. coli* CVCC 1515 cells (4 × 10^6^ CFU/mL) were incubated with 500 μL of macrophage suspension and 100 μL of baby rabbit complement (Cedarlane, Hornby, ON, Canada) at 30 °C for 1 h. Macrophages were then lysed by adding sterile water into the mixture (Rennermalm et al. 2001; Xu et al. 2011; Gressler et al. 2016). The mixture was then serially diluted for the plate count. The bacterial killing rate was calculated as the formula: [1− (number of bacteria recovered in the presence of phagocytes/number of bacteria recovered in the absence of phagocytes)] × 100% (Liu et al. 2012). Data from three independent experiments are expressed as percentage (mean ± standard deviation) of killed bacteria.

### Serum bactericidal assay

The serum bactericidal test was carried out according to pervious protocols with some modification (Maslanka et al. 1997; Marzoa et al. 2012). 12.5 μL of *E. coli* CVCC 1515 cells (5–6 × 10^3^ CFU/mL), 50 μL of serial twofold mouse serum, and 25 μL of baby rabbit complement were added into each well of a 96-well cell culture plate. Each group contained (i) bacteria (12.5 μL) + complement (25 μL) + immunized serum or unimmunized serum (50 μL, eightfold serially diluted in PBS) (complement-dependent manner) and (ii) bacteria + heat-inactivated complement + immunized serum or unimmunized serum (complement-independent manner). The plates were incubated at 37 °C for 60 min, and 20 μL of samples from each well were plated onto LB agar. After overnight incubation, the plates were counted.

All statistical analyses were performed using SPSS version 22.0. Differences were considered significant at *p* < 0.05.

## Results

### Homology analysis of OmpF

**Fig. 1:**
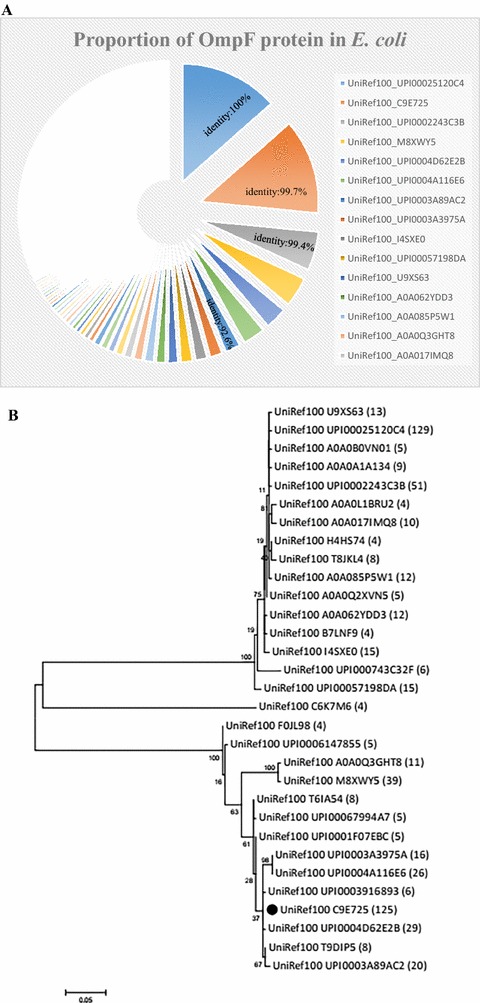
Homology and phylogenetic analysis of the OmpF protein. **A** Proportion of the OmpF protein sequences in *E. coli* (%); **B** phylogenetic analysis of the OmpF protein in *E. coli* (omit the clusters less than 4 in size). *Black symbol* indicates the protein clusters which were identical to that of *E. coli* CVCC 1515 OmpF

**Fig. 2:**
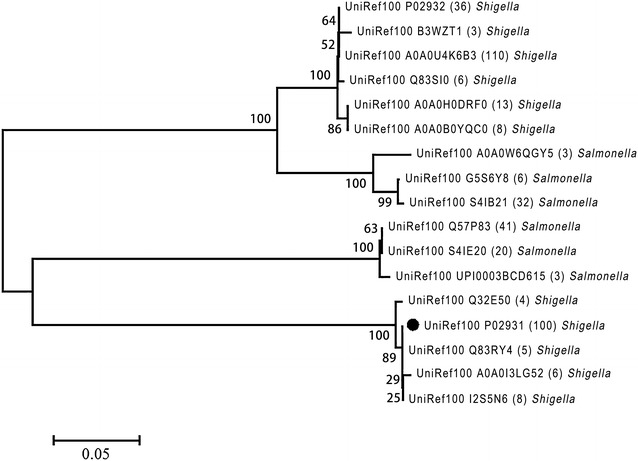
Homology and phylogenetic analysis of the OmpF protein. Phylogenetic analysis of the OmpF protein in *Shigella* and *Salmonella* (omit the clusters less than 3 in size). *Black symbol* indicates the protein clusters which were identical to that of *E. coli* CVCC 1515 OmpF

The BLAST result showed that proportion of the OmpF protein sequences in *E. coli* was up to 100% (Fig. 1A). The amino acid sequences of OmpF from *E. coli* CVCC 1515 shared 99, 100 and 100% identity among *E. coli* CICC 21530 (O157:H7), K12 and BL21, respectively. In order to predict the potential of OmpF porin as a universal vaccine against gram-negative bacteria, the homology analysis of OmpF protein clusters was carried out by MEGA5.1 software. The OmpF from *E. coli* CVCC 1515 was identical to that of C9E725 (*E. coli*) and P02931 (*Shigella*). It also shares a high identity (90–100%) with about half of the *E. coli* (46.7%) (Fig. 1B) and *Shigella* (52.8%) strains (Fig. 2). However, the identity with *Salmonella* strains was lower (59.5–85.1%), 67.2% of *Salmonella* protein clusters only share an identity of 62.4% with *E. coli* CVCC 1515 OmpF protein (Fig. 2). The results showed that the OmpF protein is highly conserved among *E. coli* and *Shigella*, and with a certain degree of homology with *Salmonella*.

### Expression and purification of the rOmpF protein

**Fig. 3:**
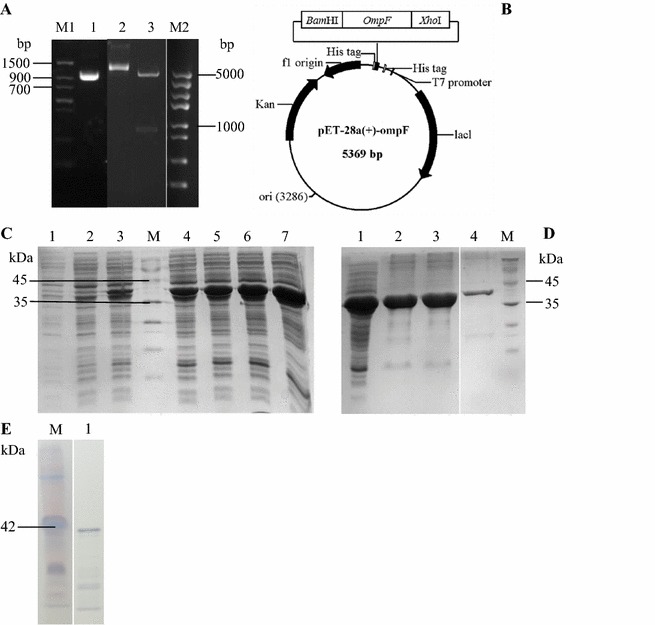
Cloning, expression, purification and Western blotting analysis of OmpF. aA PCR products of the *ompF* gene. *Lane M1 Trans* DNA Marker II (1500, 900, 700, 500, 400, 200 and 100 bp); *lane 1 ompF* PCR products. *Lane 2* the recombinant pET-28a(+)-ompF plasmid. *Lane 3* the recombinant pET-28a(+)-ompF plasmid digested with *BamH*I and *Xho*I; *lane M2 Trans*5K DNA Marker (5000, 3000, 2000, 1500, 1000, 800, 500 and 300 bp); **B** schematic representation of the pET-28a(+)-ompF plasmid; **C** SDS-PAGE analysis of the expression of rOmpF in auto-inducing media at different time. 1 mL of cultured cells were collected at 4, 6, 8, 10, 12, 14, and 24 h, respectively. *Lane M* protein marker (94.4–14.4 kDa); *lane 1* 4 h; *lane 2* 6 h; *lane 3* 8 h; *lane 4* 10 h; *lane 5* 12 h; *lane 6* 14 h; *lane 7* 24 h; **D** SDS-PAGE analysis of the purified rOmpF protein. *Lane M* 5 µL of protein marker (94.4–14.4 kDa); *lane 1* total proteins after auto-induction of *E. coli* BL21 (DE3) containing pET-28a(+)-ompF (15 µL of cell lysis); *lane 2* precipitation containing inclusion body after sonication and centrifugation (15 µL of renaturation solution); *Lane 3* flowthrough (15 µL of elution solution); *Lane 4* the eluent washed with 70% elution buffer (15 µL of the purified OmpF protein); **E** Western blotting analysis of the OmpF protein. *Lane M*, protein marker; *lane 1*, the OmpF protein

The 1023 bp *ompF* gene was successfully amplified from the genomic DNA of *E. coli* CVCC 1515 (Fig. 3A), and cloned into pMD19-T Simple vector. After being digested with *BamH*I and *Xho*I, the *ompF* fragment was inserted into a pET-28a(+) vector, and the resultant plasmid was named as pET-28a(+)-ompF. DNA sequencing result showed that the open reading frame of the *ompF* gene was composed of 1023 nucleotides and encoded a protein (341 amino acids) with a predicted molecular weight of 37.51 kDa. *ompF* shares 100% nucleotide sequence identity with that of the published *ompF* gene (GenBank Accession No. JQ886179.1), and the pET-28a(+)-ompF plasmid was successfully constructed.

As shown in Fig. 3B, the OmpF protein fused with the 6× His tag (353 amino acids) was successfully expressed in *E. coli* BL21 (DE3) by auto-inducing for 24 h at 30 °C. The molecular weight of rOmpF with an N-terminal 6× His tag was approximately 40 kDa, which matches with the expected size of 38.83 kDa. After purification by His-tag Ni affinity chromatography, the purity of the rOmpF protein was about 90%, and approximate 3 mg of the purified protein was obtained from 1 L of cultures (Fig. 3D).

### Immune response to the rOmpF vaccination

The results of Western blotting showed that the purified OmpF proteins had a main band with the size of 40 kDa (Fig. 3E). It indicated that the anti-OmpF serum mainly bound with OmpF and suggest that OmpF is an immunogenic protein.

**Fig. 4:**
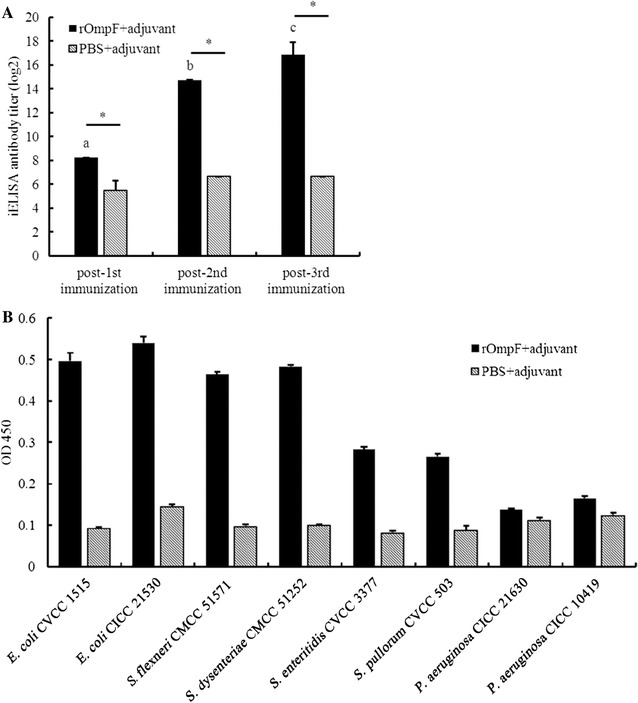
Serum responses in mice immunized with rOmpF. Mice were immunized with rOmpF at day 0 and boosted at the 3rd-week and 5th-week respectively, and sera were collected at 5-day intervals after each immunization. iELISA was used to detect the antibody titers. Results were shown as mean ± SD for 10 mice. **A** Titers of the anti-rOmpF sera against rOmpF. *Asterisk* indicates a significant difference between the OmpF immunized group and the control group (*p* < 0.05). *Different lower case letters* indicate a significant difference between each immunized (*p* < 0.05); **B** cross-reaction properties of the anti-rOmpF sera against *E. coli*, *Shigella*, *Salmonella* and *Pseudomonas*. *Y* axis indicates the optical density (OD 450) values of antibody response with different bacteria in iELISA

The anti-rOmpF sera titer was also tested by an indirect enzyme-linked immunosorbent assay (iELISA) using rOmpF as antigen. After the first immunization, the titers of anti-rOmpF against rOmpF were 1:100–1:300, and rised sharply to 1:27,000–1:240,000 after the second and third immunization (Fig. 4A). Antisera were used for following experiments after the third immunization. The antibody titers of the control mice immunized with PBS-adjuvant were only 1:30–1:100.

Additionally, the capacities for the anti-rOmpF sera binding to bacterial cells were performed among the strains of *E. coli* CVCC 1515, *E. coli* CICC 21530 (O157:H7), *S. dysenteriae* CMCC 51252, *S. flexneri* CMCC 51571, *S. enteritidis* CVCC 3377, *S. pullorum* CVCC 503, *P. aeruginosa* CICC 21630, and *P. aeruginosa* CICC 10419 in vitro. The antibody titers against *E. coli*, *Shigella* and *Salmonella* were 1:27,000, 1:27,000 and 1:9000, respectively. The OD values of 9000-fold diluted antiserum response in iELISA were shown in Fig. 4B. Antibody response of anti-rOmpF sera against *E. coli*, *Shigella* and *Salmonella* were significantly higher than the anti-PBS group. However, there was no significant discrepancy between the anti-rOmpF sera and the PBS immunized sera against *P. aeruginosa* (Fig. 4B). This indicated that the rOmpF sera strongly reacted with *E. coli*, *Shigella* and *Salmonella* strains, but not with *P. aeruginosa* strains.

### Opsonophagocytosis and serum bactericidal test in vitro

**Fig. 5:**
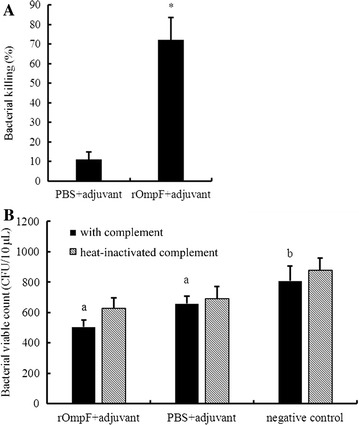
Phagocytosis and bactericidal activity of the macrophages modulated by the anti-rOmpF sera in vitro. **A** Effect of the anti-rOmpF sera on opsonophagocylic killing of *E. coli* by murine macrophages. Data are expressed as percentage (mean ± SD) of killed bacteria; **B** bactericidal activity of serum complement on *E. coli*. *Escherichia coli* CVCC 1515 was incubated for 1 h with the anti-rOmpF sera and complement (or heat-inactivated complement) (the rOmpF+ adjuvant group), the anti-PBS sera and complement (or heat-inactivated complement) (the PBS+ adjuvant group), and PBS and complement (or heat-inactivated complement) (the negative control group), respectively. Viable bacteria were counted, and data are expressed as mean ± SD. *Lower case letters* indicate a significant difference between two groups (*p* < 0.05)

Sera obtained from mice immunized with rOmpF plus adjuvant or PBS plus adjuvant were analyzed for their ability to promote opsonophagocytic killing of *E. coli* CVCC 1515 by murine peritoneal macrophages. The opsonophagocytosis result showed that only 11.04 ± 3.90% of *E. coli* CVCC 1515 could be killed in the control group (the anti-PBS serum), but 72.21 ± 11.39% of the bacteria were killed in the anti-rOmpF serum group, which suggested that the antibodies against rOmpF were effective for mediating opsonophagocytosis of *E. coli* (Fig. 5A).

The bactericidal efficiency of the complement pathway was further determined. As shown in Fig. 5B, the viable count of *E. coli* was lower in the anti-rOmpF sera group (rOmpF + adjuvant) than in the anti-PBS sera (PBS + adjuvant) and negative control groups. In the presence of both the anti-rOmpF sera and complement, the minimum number of bacterial cells were observed, indicating complement-mediated opsonic activity of the anti-rOmpF sera for *E. coli* in vitro.

### Protection efficacy after immunization with rOmpF in vivo

**Fig. 6:**
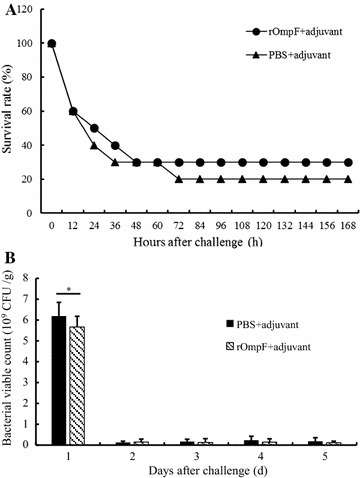
Protection efficacy after immunization with rOmpF against *E. coli* CVCC 1515 and CICC 21530 (O157:H7) in vivo. After 2 weeks of the final immunization, the mice were challenged with *E. coli* CVCC 1515 by intraperitoneal injection (**A**) or *E. coli* CICC 21530 (O157:H7) by gastric tube (**B**), and observed for 7 days after challenge. Data are expressed as mean ± SD. **A** The survival ratio of the mice immunized with rOmpF or PBS; rOmpF+ adjuvant: group 1; PBS+ adjuvant: group 2, control; **B** fecal shedding of the mice immunized with rOmpF or PBS; rOmpF+ adjuvant: group 3; PBS+ adjuvant: group 4, control. *Asterisk* indicates a significant difference between two groups (*p* < 0.05)

The immune protection efficiency of rOmpF was investigated in a murine model. After being challenged with *E. coli* CVCC 1515 within 12–36 h, the survival ratio of the rOmpF-immunized mice was decreased from 60 to 40%, but kept to 30% at 48 h postchallenge, which was higher than that of the PBS-immunized mice (control group) (Fig. 6A).

In our study, mice were administered orally with *E. coli* CICC 21530 (O157:H7), and *E. coli* in fecal shedding was examined by plate counting. As shown in Fig. 6B, a significant decrease in fecal shedding of *E. coli* was observed at 2 days postchallenge in the rOmpF-immunized mice and the PBS-immunized mice, but there was no significant difference in the bacterial count in fecal shedding between the immunized and control group at the subsequent time point. The duration of the *E. coli* shedding in mice lasted no longer than 1 day.

## Discussion

Considering the diversity of pathogenic *E. coli* serotype, we took more attention on developing a versatile vaccine that provides heterologous protection for *E. coli*, even other gram-negative pathogens such as *Salmonella* or *Shigella*. Lots of diarrheal illness were co-infected by these strains (O’Ryan et al. 2015). As we all known, porins exist most frequently as trimers and the sequence homology among porins of several genera such as *Escherichia* and *Neisseria* has shown a highly conserved nature (Yadav et al. 2014). Therefore, as a porin of *E. coli*, the OmpF protein was selected to be a candidate vaccine in our study.

Previously, we have studied the OmpA and OmpC of *E. coli*, the result showed that they all shared a high identity with some typical *E. coli*, *Salmonella* and *Shigella* strains, and possessed satisfied immunogenicity (Guan et al. 2015; Wang et al. 2015). Therefore, in this study, the homology of OmpF protein clusters in these strains were further analyzed, thus leading to a better manifestation of the sequence conservation. The result implies that OmpF may be a shared antigen among *E. coli* and *Shigella* strains and these consensus regions should be helpful in designing universal vaccines against a broad range of gram-negative pathogens.

The OmpF protein from *E. coli* has antigenic epitopes located on several extracellular loops, indicating that it may have some immune properties (Liu et al. 2012; Klebba et al. 1990; Fourel et al. 1993). In our study, the protein was expressed in the formation of inclusion bodies (IBs). In order to fully expose extracellular epitopes and then obtain better immunogenic response for the whole cell, the rOmpF was further resolubilized by denaturant (urea), and refolded by the assistant of detergent (LDAO) which could simulate the natural environment (Saleem et al. 2012).

Previous studies demonstrated that antigen proteins with higher purity, such as M2 (>90%), the culture filtrate proteins (CFP) (>96%), PfEBA-175II F2 (>95%) and Mtb72F (>98%) displayed good immunogenicity (Frace et al. 1999; Roberts et al. 1995; Zhang and Pan 2005; Skeiky et al. 2004). However, some recombinant proteins such as Bm95, E6/E7, OprF-OprI and EspA-Stx2A1 with low purity of 80–90% also display the effective immunogenicity (García-García et al. 2000; De Bruijn et al. 1998; von Specht et al. 1995; Cheng et al. 2009). In comparison, the purity of rOmpF (90%) obtained in this study falls within the scope of the above antigen proteins and meets the purity requirement of vaccine preparation.

As expected, BALB/c mice vaccinated with the renatured and purified rOmpF protein elicited a significant immunogenic response, which was consistent with the previous report (Sidorova et al. 2014). The iELISA results showed that the antiserum not only had high affinity against rOmpF (1: 240,000 dilution) but also against the whole cell (1:27,000 dilution) (Fig. 4). It was demonstrated that the rOmpF extracellular epitopes especially the conformations can restore after renaturation, some of the antibody against them can specifically recognize the bacteria. Therefore, the protein got the prerequisite for being used as a subunit vaccine. Moreover, significant antigenic cross-reactivity responses to *Shigella* and *Salmonella* strains were observed which is in accordance with that of homology analysis, indicating that rOmpF may be a potential candidate for a universal vaccine.

It was well known that antibody could mediate phagocytosis of organisms when the antibody constant region was recognized by the Fc receptors of phagocytes such as neutrophils and macrophages. Additionally, the Fc regions of antibodies maybe bind and activate complement proteins that can directly cause bacterial death (Clemens et al. 2003). All the bactericidal function implementation depends on the affinity of the Fab region with the antigenic components of bacterial pathogens, which has been proved by the iELISA results. In present study, a significant bactericidal effect was achieved in the opsonophagocytosis assay and the serum bactericidal assay (Fig. 5), indicating a phagocytosis and classical complement pathway killing mediated by the antiserum. It was consistent with the results of the previous studies (Liu et al. 2012; Wang et al. 2015; Marzoa et al. 2012; Seder and Mascola 2003). However, the role of OmpF in macrophage adherence and cytokine production needs to be further evaluated.

The porin protein not only has the ability to elicit a host immune response, but also protect the host against infection (Secundino et al. 2006). Above results demonstrated that rOmpF induced a strong immune response, but conferred no significant protection against *E. coli* CVCC 1515 in BALB/c mice, which was consistent with previous results reported by Toobak et al. (2013). Meenakshi et al. referred to an irrelevance of the increased antibody level as an indicator of a protective effect against *Salmonella* (Meenakshi et al. 1999). There are several possible reasons for lower protection in this study: (i) the characteristic of a prokaryotic expressed system cannot provide the glycosylation, which may affect the antigenicity; (ii) the native active configuration of rOmpF after denaturation and renaturation could be altered, formation of a partially folded or misfolded conformation, which may affect the immunogenicity (Dowling et al. 2007); and (iii) the anti-rOmpF sera did not reach or recognize the OmpF protein on the outer membrane of live *E. coli* due to the presence of lipopolysaccharide (LPS), pili, flagella, and other porin proteins, which can mask the OmpF protein (Okamura et al. 2012). It was reported that OmpF and OmpC are tightly bound to the LPS (Fourel et al. 1993). This viewpoint was supported by other researchers, who suggested that the potential limitation of the interaction between an antibody and OMPs of live bacteria by O-chain of LPS (Singh et al. 2003). Tarkka et al. also pointed out that it is difficult for the anti-OMP sera to reach OMP on live *N*. *meningitides* (Tarkka et al. 1989). Further increasing the yields of correctly folded rOmpF by addition of hydrophobic agents such as ethanol, DMSO (dimethylsulfoxide), and acetonitrile to the renaturation buffer and further study using different vaccination methods, including antigen dose, times, adjuvant and more mice need to be elucidated.

OMPs in gram-negative bacteria have been reported to induce the host humoral responses, and in turn inhibit postchallenge bacterial colonization (Okamura et al. 2012). Therefore, a preliminary evaluation of the wide spectrum of the rOmpF vaccine was carried out. Though the high bacterial count in fecal shedding was only last 1 day, which is shorter than that of the previous study (Fan et al. 2012), this difference may be due to the variation of strains that causes the antigenic variation. However, the microbe amount of the immunized group was significantly decreased before excreting from the bowel (Fig. 6B). It is suggested that by induction of a strong immune response, rOmpF conferred a cross protection against *E*. *coli* in BALB/c mice to a certain degree.

In conclusion, the expressed OmpF protein induced an effective immune response, including the high antibody titer and high affinity of the antibody that binds to the bacterial surface. Additionally, rOmpF increased the capacity of macrophages to clear bacteria, and it was found for the first time that the anti-rOmpF sera had a significant cross-reaction capacity against *Escherichia*, *Shigella*, and *Salmonella* strains in vitro. Although rOmpF only provide a weak protection against *E. coli* and reduce *E. coli* in fecal shedding in vivo, we believe OmpF in native forms might be suitable as a vaccine candidate.

## Abbreviations

OmpF: outer membrane protein F
rOmpF: recombinant OmpF
OMPs: outer membrane proteins
IgG: immunoglobulin G
iELISA: indirect enzyme-linked immunosorbent assay
LPS: lipopolysaccharide
DMSO: dimethylsulfoxide
CVCC: China Veterinary Culture Collection Center
CMCC: National Center for Medical Culture Collection
CICC: China Center of Industrial Culture Collection
EDTA: ethylenediamine tetraacetic acid
HRP: horseradish peroxidase
TMB: 3, 3′, 5, 5′-tetramethylbenzidine
CAAS: Chinese Academy of Agricultural Sciences
IBs: inclusion bodies
SDS-PAGE: sodium dodecyl sulfate polyacrylamide gel electrophoresis
